# FcRn receptor-mediated pharmacokinetics of therapeutic IgG in the eye

**Published:** 2009-12-16

**Authors:** Hyuncheol Kim, Shaun B. Robinson, Karl G. Csaky

**Affiliations:** 1 Department of Chemical and Biomolecular Engineering, Sogang University, Seoul, South Korea; 2 Department of Ophthalmology, Duke University Medical Center, Durham, NC

## Abstract

**Purpose:**

The goal of this study was to determine the role of the neonatal Fc (FcRn) receptor in eliminating intravitreally administered full-length immunoglobulin G (IgG) across the blood-retinal barrier.

**Methods:**

FcRn receptor expression in normal and laser photocoagulated retinas was compared quantitatively by real-time RT–PCR. The distribution of intravitreally administered full-length IgG was investigated and compared in wild-type and FcRn knockout mouse eyes as well as normal and laser-photocoagulated rat eyes at several time points. Additionally, the pharmacokinetics of intravitreally injected full-length IgG and chicken immunoglobulin Y (IgY) was compared in the normal rat retina.

**Results:**

Intravitreally administered full-length IgG overcame the inner limiting membrane and diffused into the deeper retinal structures in both normal and laser-photocoagulated retinas. Interestingly, IgG was eliminated across the blood-retinal barrier into the blood system in the normal retina, whereas IgY was not. In addition, full-length IgGs did not penetrate across the blood-retinal barrier in the FcRn knockout mouse. Intravitreally injected IgGs were eliminated into the blood system more rapidly in laser-photocoagulated eyes when compared to normal control eyes because of FcRn receptor upregulation in the laser-photocoagulated retina.

**Conclusions:**

FcRn plays an important role in eliminating intravitreally administered full-length IgGs across the blood-retina barrier into the systemic blood system.

## Introduction

Interest in antibody-based treatment for retinal disorders has recently increased with the availability of efficacious and FDA-approved antibodies. To date, more than 20 antibodies have been approved by the United States Food and Drug Administration (FDA) for therapeutic use in humans [1]. At least five humanized monoclonal antibodies are being tested in more than ten ocular clinical trials (March 2009). Direct intravitreal injection has become a common approach for delivering therapeutic antibodies to the posterior segment of the eye for retina disorders. Although intravitreally injected full-length antibodies penetrate the retina as easily as antibody fragments [2], the fate of these antibodies after they access the retina is not yet understood.

The inner blood-retinal barrier plays an important role in supplying oxygen and nutrients to the retinal cells, not unlike that performed by the blood-brain barrier [3]. It is formed by complex tight junctions on the retinal capillary endothelial cell, which are themselves further enveloped with pericytes and Müller cells [3]. Thus, the blood-retinal barrier is structurally similar to the blood-brain barrier [4]. Several influx and efflux transporters have been identified and characterized in brain capillary endothelial cells [3,5]. Schlachetzki et al. [6] determined that the blood-brain barrier contains the neonatal Fc (FcRn) immunoglobulin G (IgG) receptor/transporter [6]. Efflux of intracerebral IgG is FcRn-mediated as intracerebral IgG elimination into the systemic circulation is competitively inhibited by Fc fragments, which block the FcRn receptor, but not impeded by Fab fragments, which do not bind to the FcRn receptor [7,8]. In addition, Deane et al. [9] concluded that the FcRn pathway at the blood-brain barrier plays a crucial role in IgG-associated amyloid beta peptide removal from the aging brain [9]. In prior studies, we have established the expression of FcRn at the blood-retinal barrier [10]. Therefore, in this study, we investigated the trans-retinal penetration and FcRn-dependent elimination of intravitreally administered IgG across the blood-retinal barrier.

## Methods

### Animals

Male adult (250 g) Brown Norway rats (Charles River Laboratories, Raleigh, NC) was used in the study. All the animals were housed under a 12 h light-dark cycle with standard diet and free access to water ad libitium. All procedures adhered to the guidelines from the Association for Research in Vision and Ophthalmology for the Use of Animals in Research.

### Intravitreal injection of fluorescence labeled antibody in rats

Two ml of Alexa 555 conjugated goat anti-rabbit IgG (GAR555; H+L; Invitrogen, Carlsbad, CA) were dialyzed two times overnight with a dialysis cassette (MWCO=10 kDa; Pierce, Rockford, IL) in 1,000 ml of 1× phosphate-buffered saline (PBS, 137 mM NaCl, 2.7 mM KCl, 10 mM Na_2_HPO_4_, 1.8 mM KH_2_PO_4_, pH 7.4) to facilitate removal of dissolved sodium azide. And then, the dialyzed antibody solution was centrifugated at 16,873× g for 5 min at 4 °C to remove antibody aggregate. Rats aged 8–12 weeks were anesthetized by intramuscular injection of 70 mg/kg ketamine and 30 mg/kg xylazine before both pupils were dilated with 10% tropicamide (Mydriacyl; Alcon, Fort Worth, TX). Next, 5 μl of the aforementioned antibody solution (2 mg/ml) was injected intravitreally into the right eye with a 32-gauge needle. After CO_2_ euthanasia, the injected eyes were then enucleated at either 30 min, 6 h, or 15 h post injection, respectively. The enucleated eyes were immediately immersed in a 4% paraformaldehyde (PFA) solution and held at 4 °C overnight. The fixed eyes were then placed in 1× PBS at 4 °C until the next experiments. To determine the distribution of intravitreally injected antibody in a post-mortem eye, we immediately injected 5 μl of GAR555 (2 mg/ml) after CO_2_ euthanasia. The eye was enucleated, kept in 1× PBS for 6 h at 4 °C and then fixed overnight with 4% PFA solution at 4 °C. As described, the eyes were stored in 1× PBS at 4 °C until the next steps.

### Retinal laser photocoagulation model in rats

To determine the distribution of intravitreally injected antibody in a laser photocoagulated retina, we created a laser-photocoagulated retina. Animals were anesthetized as described in the preceeding paragraph. The right eye was dilated with 1% tropicamide. Hydroxypropyl methylcellulose (DECA Pharmaceuticals, LLC, Bowling Green, KY) was applied to each eye, and a microscope slide cover glass was used as a contact lens. The right eyes each received nine laser burns (3×3 grid pattern) between retinal vessels using the blue-green setting of a Lumenis Ultima Argon Laser (Lumenis Inc., New York, NY). The laser power was 180 mW for 0.1 s with a 50 µm spot diameter. The retina was examined to confirm no bleeding had occurred. Two weeks later, 5 µl of GAR555 (2 mg/ml) was injected as described. The eyes were then enucleated at either 1 h or 3 h post injection before immediate fixation in 4% PFA solution at 4 °C overnight. The fixed eyes were stored in 1× PBS at 4 °C until the next experiments. The optic nerve and conjunctival tissues above the sclera were carefully removed and the fixed eye was opened by a circumferential incision at the surgical limbus. The anterior segment and lens were removed, and the retina was gently separated from the sclera tissue. The retina was embedded in 7% agarose type XI gel (Sigma, St. Louis, MO) and sectioned into 100 µm-thick slices with a vibrating blade microtome (Leica, Bannockburn, IL). The eye sections were incubated overnight in 1:1,000 DAPI solution diluted with ICC buffer (PBS containing 0.5% BSA, 0.2% Triton X-100, and 0.05% sodium azide, pH 7.4) and then washed for 3 h three times with ICC buffer. Finally, the eye sections were embedded in mounting medium (Vectashield, Vector Laboratories, Inc., Burlingame, CA) and sealed under microscope cover glass slides before being viewed on a Leica laser scanning confocal microscope.

### Expression of FcRn receptor in the laser-photocoagulated retina of rats

The FcRn mRNA level in laser photocoagulated retinas was compared with that in normal retinas using the following technique. Animals received 20 laser burns in each eye. Retinal tissue (each group n=4) was carefully isolated from the enucleated eyes immediately after CO_2_ euthanasia. Total RNA was then isolated and purified from the retinal tissue with the TRIzol plus RNA purification kit (Invitrogen) according to the manufacturer’s protocol. The first-strand cDNA was synthesized from 2 µg of total RNA with SuperScript III first-strand synthesis system for RT–PCR (Invitrogen) following the manufacturer’s protocol. The resulting first-strand cDNAs were stored at −80 °C before use. Aliquots of cDNA were subjected to PCR reaction using the following custom-made amplification [11] primers 5′- CTG TGG ATG AAG CAA CCT G-3′ and 5′-TCC ACG TTT GAC CTC TAG C-3′ (Integrated DNA Technologies, Coralville, IA).

### Distribution of intravitreally injected bevacizumab in the FcRn knockout and wild-type mice

We injected 1 µl bevacizumab (25 mg/ml) intravitreally in FcRn knockout mice (B6.129X1-Fcgrt^tm1Dcr^/Dcr) and wild-type mice (C57BL/6J) were purchased from Jackson Laboratory (Bar Harbor, ME). All mice were housed at specific pathogen-free facilities. Water and food were provided ad libitum. The eyes were enucleated 5 h post injection and fixed immediately with 4% PFA solution. The fixed eye was opened by a circumferential incision at the surgical limbus. After the anterior segment of the eye and lens was removed, the retina was carefully separated from the sclera tissue. The retina was embedded in 7% agarose type XI gel (Sigma) and sectioned into 100 μm-thick slices with a vibrating blade microtome (Leica). The bevacizumab (recombinant humanized IgG1) in the retina was determined with Alexa 488 conjugated goat anti-human IgG. The isolated retinas were incubated overnight in 1:1,000 DAPI and 1:200 Alexa 488 conjugated goat anti-human IgG diluted with ICC buffer. Retinas were then washed for 3 h three times with ICC buffer. The eye sections were embedded in mounting medium (Vectashield) and sealed under microscope cover glass slides. The eye sections were viewed on a laser scanning confocal microscope and the fluorescence intensities across the retinal capillaries were determined with Image J (version 1.49 g; National Institutes of Health, Bethesda, MD). Finally, these fluorescence intensity values were normalized using the maximum intensity value at the abluminal side of the retinal capillary as the normalizing value.

### Pharmacokinetics of intravitreally injected bevacizumab in the laser photocoagulated eye of rats

Two weeks after the rats were given their laser burns, both lasered and non-lasered age-matched control animals were anesthetized using the same anesthesia technique described in the previous section. Additionally, left eye analgesia and pupillary dilation were achieved with 1 drop of 0.5% proparacaine hydrochloride (Alcon, Barrio Palmas, Puerto Rico) and 1 drop of 2.5% phenylephrine hydrochloride (Akron, Inc., Decatur, IL), respectively. The left eye was then examined using methylcellulose and a microscope slide coverslip to confirm CNV within the retina of the laser photocoagulated animals. In both the laser photocoagulated and non-lasered control groups, the left retina was subsequently injected intravitreally roughly 1 mm behind the surgical limbus in the superotemporal quadrant with 2 µl bevacizumab at 25 mg/ml (50 μg) using a 30-gauge syringe (Hamilton, Reno, NV). Five hours later, blood samples were obtained intracardially from the animals and then the animals were euthanized with CO_2_. Serum samples were immediately collected from the blood samples.

### Bevacizumab enzyme-linked immunosorbent assay

Bevacizumab concentrations in serum samples were quantified by enzyme-linked immunosorbent assay (ELISA). All steps were performed at room temperature. A Microlite2+high-binding 96-well plate (Thermo Fisher Scientific, Waltham, MA) was coated with goat anti-human IgG affinity purified antibody (Bethyl Laboratories, Montgomery, TX) diluted to 10 µg/ml in coating buffer (0.05 M carbonate-bicarbonate, pH 9.6). After 1 h incubation, each well was washed three times with 200 µl of washing solution, which contained 50 mM Tris, 0.14 M NaCl, and 0.05% Tween-20, pH 8.0. Samples were then blocked for 30 min with 200 µl of blocking solution, which contained 50 mM Tris, 0.14 M NaCl, and 1% BSA, pH 8.0. Each well was then washed three times with 200 µl of washing solution.

Samples were diluted 1:100 in sample diluent, which contained 50 mM Tris, 0.14 M NaCl, 1% BSA, and 0.05% Tween-20, pH 8.0. Approximately 100 µl of diluted sample were aliquoted into each well, and samples were incubated for 1 h. After the incubation, each well was washed five times with 200 µl of washing solution. Next, 100 µl of biotin-labeled Fc fragment specific goat anti-human IgG with minimal reactivity to Mouse IgG (eBioScience, San Diego, CA) was diluted to 20 µg/ml in the previously described sample diluents and added to each well and samples were incubated for 1 h before being washed with the washing solution. Afterwards, 100 µl of Alexa Fluor 488 conjugated streptavidin, which had been diluted to 10 µg/ml in the sample diluents was added to each well, and samples were incubated for 1 h before being washed with the washing solution. Finally, after 100 µl of PBS was added to each well, the plate was read at an excitation of 485 nm and emission of 525 nm. The standard curve was obtained by serial dilutions of a 25 mg/ml bevacizumab solution (Genentech Inc., San Francisco, CA). The bevacizumab concentration in each diluted serum sample was determined from the standard curve using linear regression. The bevacizumab concentration of these diluted samples was then used to calculate the original, undiluted bevacizumab concentration in the serum.

### Pharmacokinetics of intravitreally injected bevacizumab and chicken IgY in rats

Rats were given intravitreal injections of 2 µl of either 5.7 mg/ml bevacizumab or 5.7 mg/ml chicken IgY (Jackson ImmunoResearch Laboratories, Inc., West Grove, PA). Then 5 h post injection, the eyes were enucleated after CO_2_ euthanasia and immediately fixed in 4% PFA. Next, 10 μm-thick frozen sections of either bevacizumab- or chicken IgY-injected eyes were prepared as described. The distribution of either bevacizumab or chicken IgY in the frozen sections was determined with Alexa dye conjugated goat anti-human IgG or Alexa dye conjugated goat anti-chicken IgG, respectively.

## Results

### Intravitreally administered full-length antibody distribution in the rat retina

**Figure 1:**
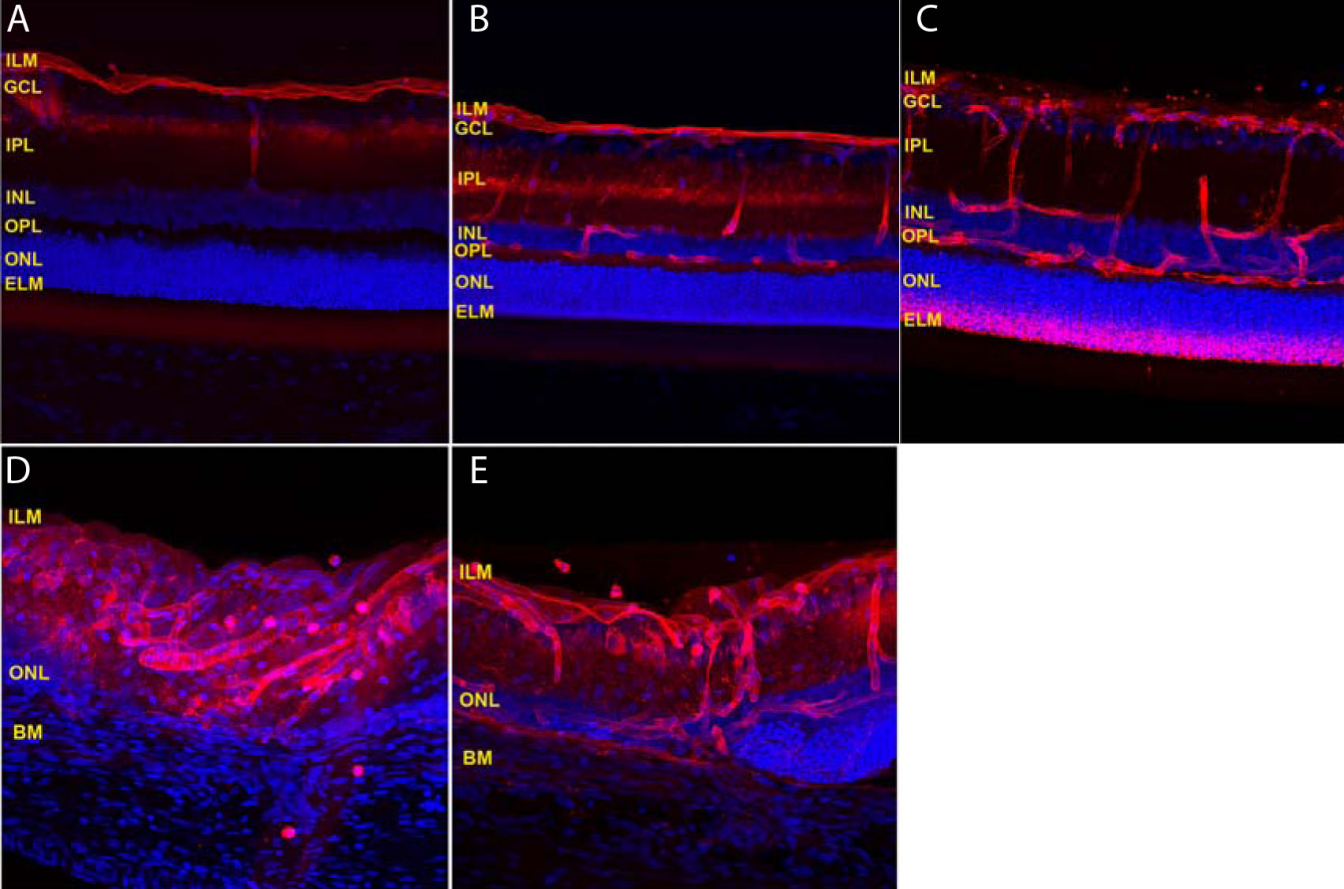
The distribution in rat eyes of intravitreally administered fluorescent dye labeled full-length IgG (red). **A**-**C** show the distribution of the full-length IgG in the normal retina at 30 min, 6 h, and 15 h post intravitreal administration, respectively. **D** and **E** show the distribution of the full-length IgG in the laser-photocoagulated retina at 1 h and 3 h post intravitreal administration, respectively. Intravitreally administered full-length antibody overcame the inner limiting membrane barrier and diffused into the deeper retinal structures in both laser photocoagulated and control rat retinas. **A**–**E**: Cell nuclei were stained with DAPI (blue). Abbreviations: inner limiting membrane (ILM), ganglion cell layer (GCL), inner plexiform layer (IPL), inner nuclear layer (INL), outer plexiform layer (OPL), outer nuclear layer (ONL), external limiting membrane (ELM), and Bruch’s membrane (BM).

Intravitreally administered full-length antibody overcame the inner limiting membrane barrier and diffused into the deeper retinal structures in both laser-photocoagulated and non-lasered rat retinas (Figure 1A-C). The antibody did not penetrate the external limiting membrane but, instead, actually accumulated along the external limiting membrane in non-lasered retinas (Figure 1C). On the other hand, the antibody easily penetrated the retina into the subretinal layer and Bruch’s membrane region in laser photocoagulated retinas (Figure 1D-E). Figure 1 demonstrates the prevalence of IgG associated with the retinal capillaries in both normal and laser-photocoagulated retinas.

**Figure 2:**
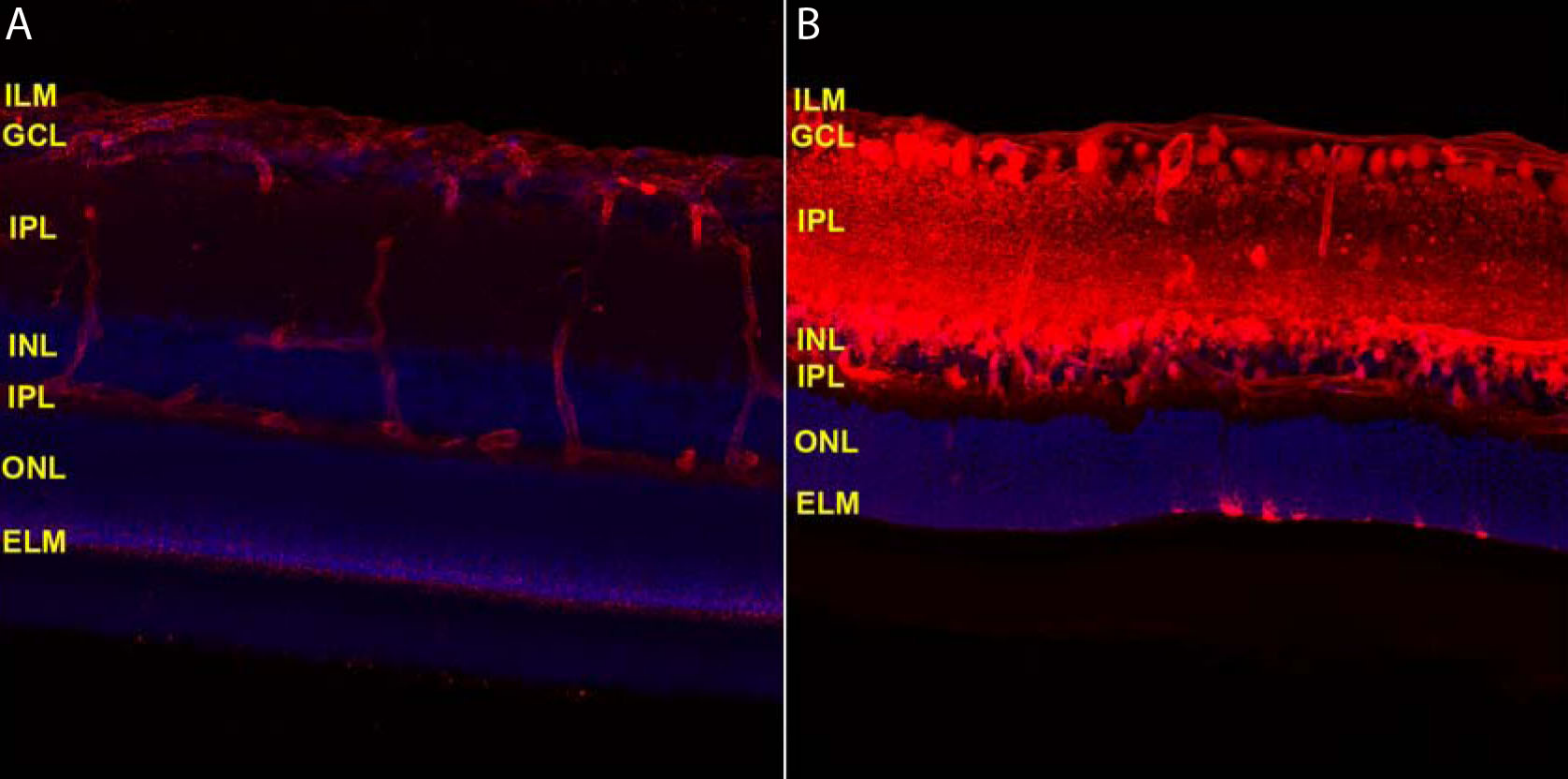
The distribution in rat eyes of intravitreally administered fluorescent dye labeled full-length IgG (red). **A** and **B** show the distribution of the full-length IgG in the live and post-mortem retina 6 h post intravitreal administration, respectively. More antibodies were observed in the post-mortem retina, compared to the live retina, suggesting the presence of active elimination mechanisms for IgG in the live retina. **A**, **B**: Cell nuclei were stained with DAPI (blue). Abbreviations: inner limiting membrane (ILM), ganglion cell layer (GCL), inner plexiform layer (IPL), inner nuclear layer (INL), outer plexiform layer (OPL), outer nuclear layer (ONL), and external limiting membrane (ELM).

Figure 2A,B demonstrate injection of full-length IgG antibody intravitreally to a live eye and a postmortem eye to see the different distribution of the injected antibody in the live and postmortem retinas, respectively. More antibodies were observed in the postmortem retina, compared to the live one, which strongly suggests the presence of active elimination mechanisms for IgG in the live retina.

### The pharmacokinetics of intravitreally injected full-length IgGs in the laser-photocoagulated rat eye

**Figure 3:**
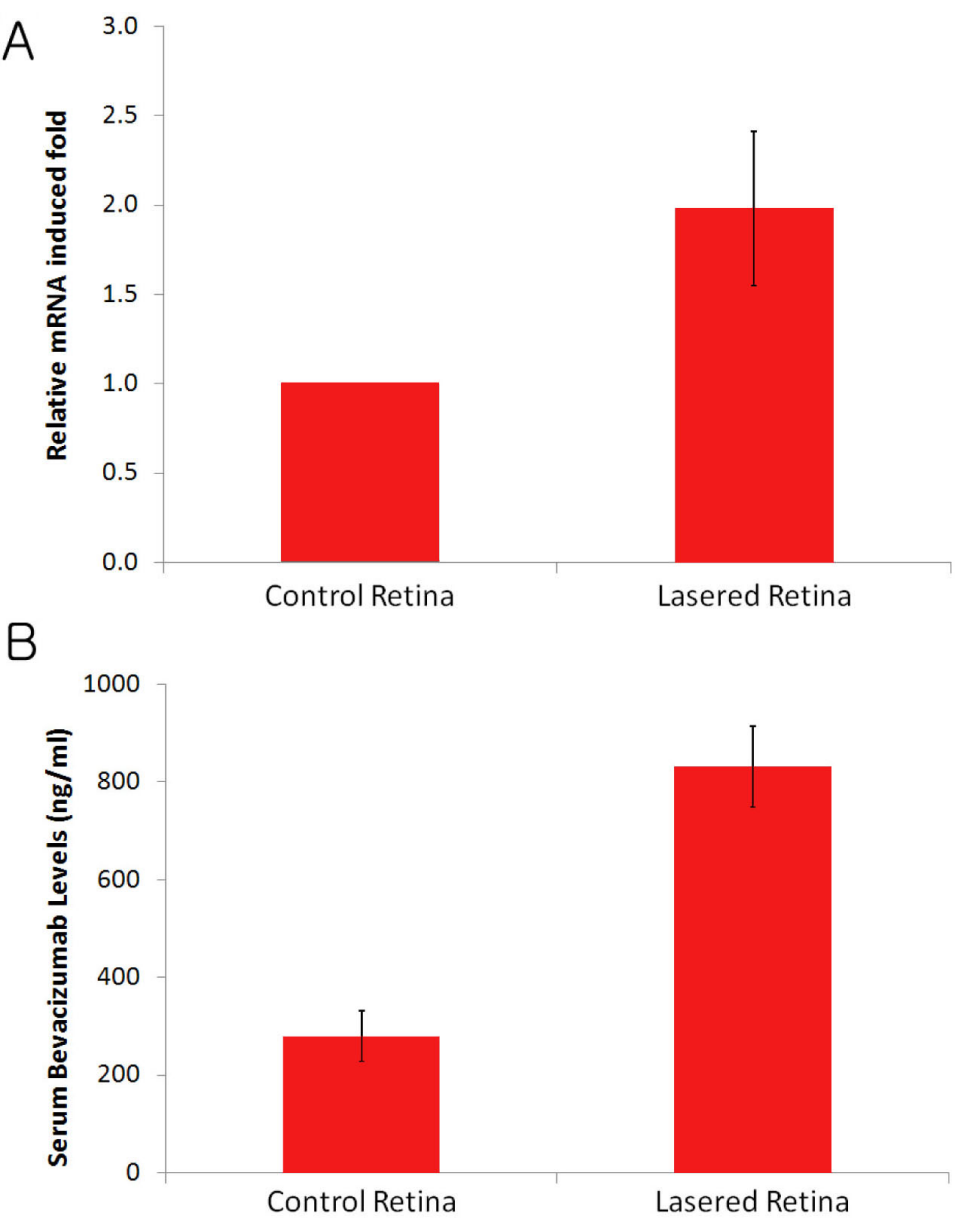
Correlation between the expression of FcRn receptor and the serum concentration of intravitreally administered bevacizumab. **A**: Real-time RT–PCR study in rats demonstrated a 1.82-fold upregulation of FcRn mRNA expression in laser-photocoagulated retinas, relative to normal retinas (n=5, p=0.001). **B**: The serum concentration of bevacizumab in the laser-photocoagulated group (832 ng/ml) was higher than that in the control group (279 ng/ml) 5 h post intravitreal administration of 2 µl bevacizumab at 25 mg/ml (n=4; p<0.05). The error bars are standard error of the mean.

Real-time RT–PCR study demonstrated a 1.82-fold upregulation of the FcRn receptor in laser-photocoagulated retinas, relative to non-lasered age-matched control retinas (p<0.05) (Figure 3A). Moreover, the serum concentration of bevacizumab 5 h post intravitreal injection was 279 ng/ml in the control group and 832 ng/ml in laser-photocoagulated group, respectively (p<0.05; Figure 3B). Accordingly, laser photocoagulation of the retina upregulated FcRn expression and resulted in a more rapid elimination of IgG from the eye into the systemic circulation.

### Intravitreally administered full-length IgG distribution in FcRn knockout and wild-type mouse eyes

**Figure 4:**
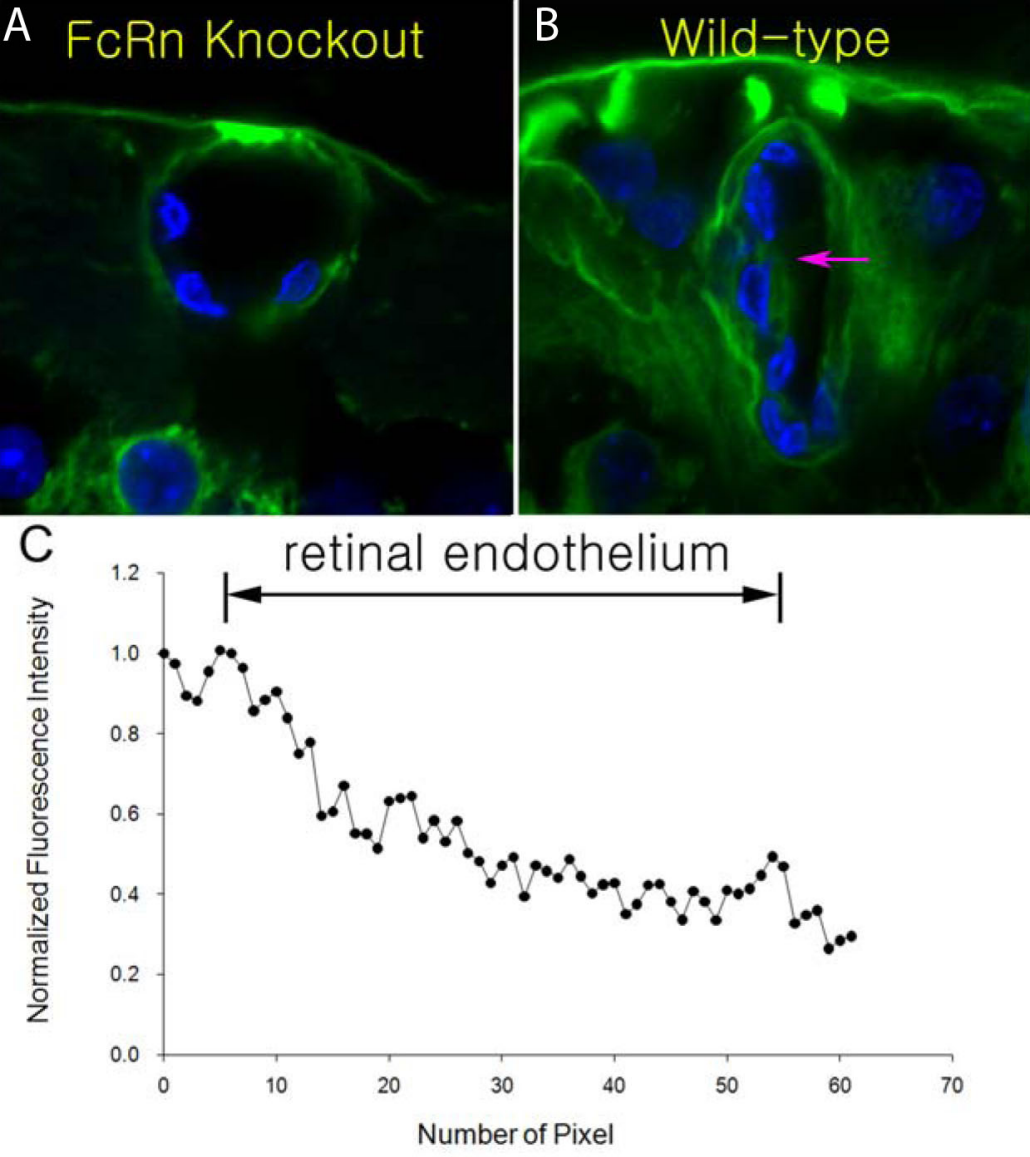
Distribution of intravitreally injected bevacizumab in the FcRn knockout and wild-type mice. **A** and **B** show the distribution of intravitreally administered bevacizumab (green) around retinal blood vessels in an FcRn knockout mouse and wild-type mouse 5 h post injection, respectively. **C** shows the normalized fluorescent intensity across the retinal vascular endothelium in a wild-type mouse. Arrows in **B** indicate the direction over which the fluorescence profiles were obtained. The normalized fluorescence intensity profile in panel **C** shows a fluorescence intensity gradient across the retinal vascular endothelium from the abluminal to luminal sides, indicating the penetration of bevacizumab into the blood system in the wild-type mouse. **A**, **B**: Cell nuclei were stained with DAPI (blue).

Figure 4 shows and compares the distribution of intravitreally administered bevacizumab, a full-length IgG, around retinal blood vessels in an FcRn knockout mouse and wild-type mouse 5 h post-injection. Interestingly, bevacizumab was only observed at the abluminal side of retinal blood vessels in the FcRn knockout mouse (Figure 4A). However, bevacizumab was detected not only abluminally but also within the lumen of the retinal blood vessels in wild-type mouse retina (Figure 4B). Moreover, it was even detected inside the vascular endothelial cells of wild-type mouse retinal blood vessels. Figure 4C shows the normalized fluorescence intensity profile gradient across the retinal endothelium, demonstrating the penetration of bevacizumab through the retinal vascular endothelium into the blood system in the wild-type mouse.

### Pharmacokinetics of intravitreally injected bevacizumab (IgG) and chicken IgY in the rat eye

**Figure 5:**
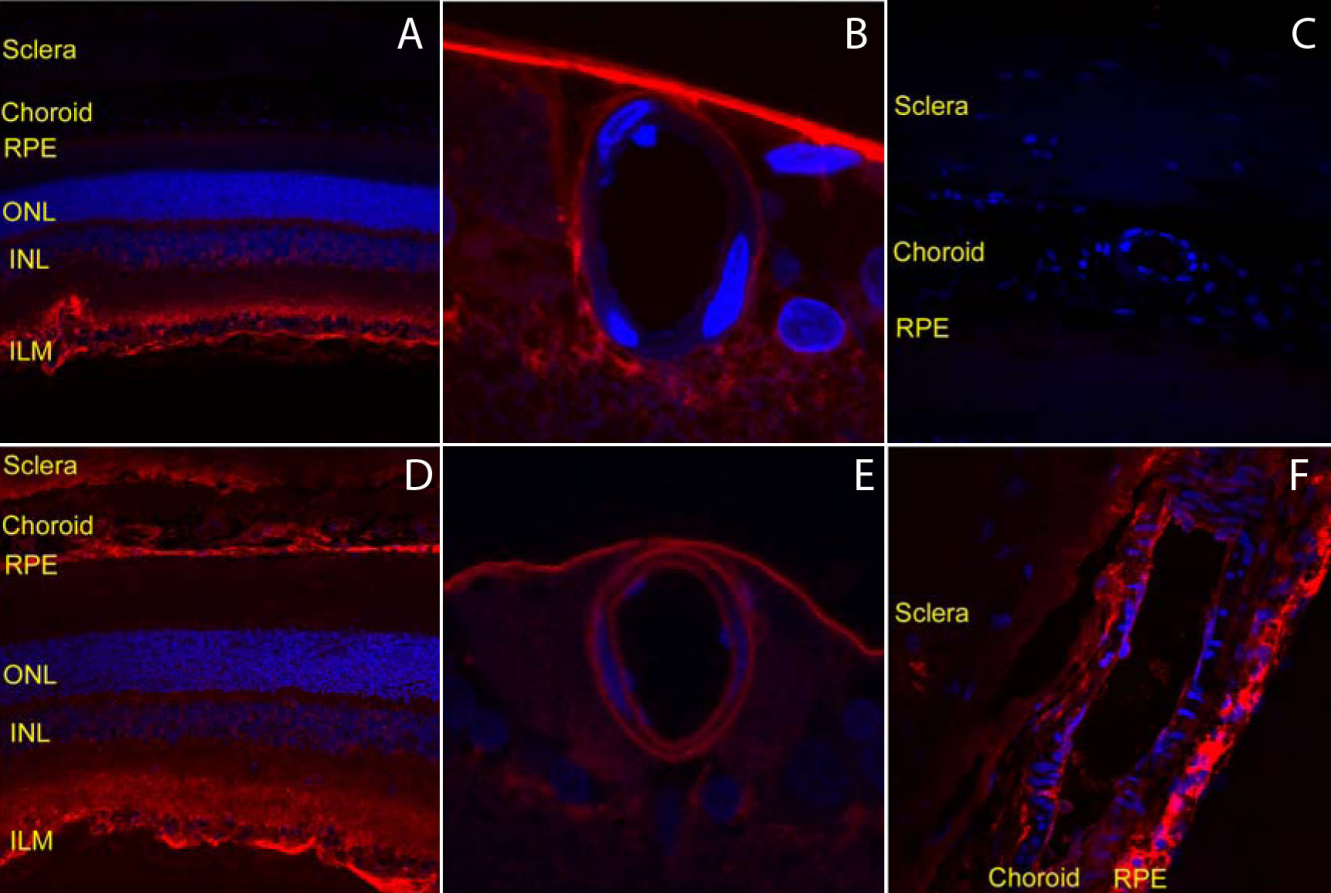
Pharmacokinetics of intravitreally injected bevacizumab and chicken IgY in rats. **A**–**C** show the distribution in rat eyes of chicken IgY in the whole retina (**A**), around the retinal blood vessel (**B**), and in the choroid (**C**) 5 h post intravitreal injection as determined by Alexa dye labeled antibodies (red). **D**–**F** show the distribution in rat eyes of bevacizumab in the whole retina (**D**), around the retinal blood vessel (**E**), and in the choroid (**F**) 5 h post intravitreal injection as determined by Alexa dye labeled antibodies (red).

Figure 5 compares the retinal distribution of IgG and chicken IgY, which is structurally similar to IgG but does not bind to the FcRn receptor [12]. Both intravitreally administered bevacizumab (IgG) and chicken IgY overcame the inner limiting membrane barrier and diffused into the deeper retinal structures (Figure 5A,D). Figure 5E demonstrates that after diffusing through the retina bevacizumab crossed the blood-retina barrier and leaked into the systemic circulation, thus explaining why bevacizumab is observed within the choroidal vasculature in Figure 5F. The intraretinal chicken IgY was only localized along the abluminal side of the blood-retina barrier (Figure 5B). Furthermore, the choroidal blood vessels were negative for the presence of chicken IgY (Figure 5C).

## Discussion

Several previous studies have reported that trans-retinal penetration varies with the molecular weight of the diffusing agent [13-16]. An in vitro study suggested the maximum molecular size capable of freely diffusing across the human retina was approximately 76.5 kDa [17]. One possible explanation for why our in vivo data (Figure 1) differs from previous in vitro studies may be the resultant structural changes in retinal integrity that inevitably occur with tissue fixation. Fixation involves cross-linkage of amino groups, causing a shrinkage of tissues and ultimately an alteration in the diffusion channels within these tissues. Furthermore, active transport mechanisms present in vivo are not present in fixed in vitro tissues. Therefore, in vitro results may be of limited utility for explaining in vivo experimental data, much less predicting clinical pharmacological phenomena observed in patients.

Figure 1A-C demonstrates that the inner limiting membrane provided a limited degree of resistance against the penetration of full-length antibody at early time points. The inner limiting membrane is composed of a fine three-dimensional meshwork structure, the pore diameter of which varies between 10 and 25 nm [18]. Stokes radius of full-length IgG is around 5.5 nm [19]. Accordingly, a 5.5-nm full-length antibody could diffuse through the inner limiting membrane into the deeper retinal structures with time. Although the inner limiting membrane acts as a filter between the retina and vitreous cavity, it is a poor barrier against the penetration of intravitreal full-length IgG into the retina.

Figure 1C shows that intra-retinal full-length IgG accumulated along the external limiting membrane in normal retinas. The external limiting membrane is composed of a row of zonulae adherents with a pore radius between 3.0 and 3.6 nm that effectively joins photoreceptors and Müller cells [14,20]. This physically smaller pore size compared to the Stokes radius of full-length IgG explains the absence of bevacizumab within the retinal pigment epithelium (RPE) and photoreceptor cell layer in a normal retina following intravitreal injection. Conversely, laser photocoagulation disrupts the external limiting membrane and extends the outer nuclear layer into Bruch’s membrane and the choroid [21,22]. So, the intravitreal administration is able to deliver full-length antibody into the subretinal space and Bruch’s membrane region because of the resultant changes in retinal morphology that occur with CNV. This explains the clinical utility of intravitreal bevacizumab against age-related macular degeneration-associated choroidal neovascularization.

The predominant elimination pathways for compounds leaving the vitreous are either into the anterior segment or across the retina. Moreover, prevailing dogma regarding the elimination route of intravitreal compounds suggests that the relative contribution of each route depends solely on the physicochemical properties of a given compound (e.g., lipophilicity and molecular size). For example, lipophilic compounds such as triamcinolone appear to be eliminated predominantly across the retina, while large, hydrophilic compounds primarily diffuse into the aqueous humor before being eliminated through conventional aqueous humor outflow pathways with the anterior chamber (trabecular meshwork and Schlemm’s canal) [23,24]. However, are physicochemical properties alone enough to explain the pharmacokinetics of intravitreally administered compounds? The physicochemical properties of fluorescein (MW=376 Da, hydrophilic) and fluorescein glucuronide (MW=508 Da, hydrophilic) are similar. The diffusion coefficients of these compounds in the aqueous phase are essentially equal (6×10^−6^ cm^2^s^−1^) [25]. Nevertheless, it is well known that the pharmacokinetics of fluorescein and fluorescein glucuronide in the vitreous are actually quite different. Fluorescein is eliminated across the retina while fluorescein glucuronide is eliminated into the anterior segment [26]. The starkly different elimination patterns of these two compounds cannot be explained solely on physicochemical properties. Instead, one must also account for the differential effect of biologic relevant transport mechanisms. Vitreal clearance of fluorescein glucuronide is largely governed by passive diffusion, while, the vitreal elimination of fluorescein is largely dependent on active transport [27]. As documented in the literature [2], the current study confirmed that intravitreally administered bevacizumab is able to easily and rapidly penetrate the retina before being eliminated into the blood circulation across the inner blood retina barrier. Additionally, intravitreal antibody is eliminated both across the iris vascular endothelial and ciliary body nonpigment epithelial tight junctions into the blood system and through conventional aqueous humor outflow pathways [28]. Finally, we documented physiologically relevant serum levels of bevacizumab after intravitreal administration, representing up to 30% of the injected dose. Of concern, this suggests greater risk for systemic side effects than previously recognized [29]. Of note, in the rabbit five-day half-life of bevacizumab within the vitreous was only slightly longer than the vitreous half-life of ranibizumab (the Fab fragment of bevacizumab), which is much less than would be expected for a molecule threefold larger than ranibizumab [30-32]. Taken together, the data indicate the blood-ocular barrier manifests a specific mechanism for transporting and clearing full-length IgGs into the systemic circulation. The current study confirms our hypothesis that this mechanism is the neonatal Fc receptor.

Anti-vascular endothelial growth factor (VEGF) antibodies, such as bevacizumab, are the first-line treatment for choroidal neovascularization in age-related macular degeneration patients. Accordingly, it is imperative from a clinical standpoint for the intraocular pharmacokinetics of full-length IgG to be established. If choroidal neovascularization significantly alters the vitreous elimination of IgG, the frequency with which patients receive intraocular IgG injections should be tailored to the level of choroidal neovascularization–just as patients with hepatic or renal dysfunction are dosed based upon their level of organ impairment. This is a novel and, as yet, unexplored area of ophthalmology. In this study, we evaluated whether animals exhibiting choroidal neovascularization had increased clearance of full-length IgG from the eye. Figure 3 demonstrates both upregulation of the FcRn receptor and faster elimination of intravitreally injected bevacizumab into the systemic circulation in the choroidal neovascularization animal model with respect to age-matched control animals without choroidal neovascularization. The pathogenesis of choroidal neovascularization is complex, involving RPE alterations, Bruch’s membrane rupture, angiogenesis, and vasculogenesis–all of which are all strongly influenced by inflammatory mediators [33]. While it is well described that VEGF triggers many of the vascular changes underlying choroidal neovascularization, new evidence suggests tumor necrosis factor α (TNF-α) is not only increased in the laser photocoagulation choroidal neovascularization model (4–5 fold) but actually contributes to the development of choroidal neovascularization [33,34]. Injection of either etanercept (soluble TNF-α receptor) or a monoclonal anti-TNF-α antibody (infliximab), each of which decrease effective levels of TNF-α, reduce the size and leakage of laser-induced choroidal neovascularization. Moreover, TNF-α upregulates both FcRn expression and function, as demonstrated by increased IgG transport across an epithelial monolayer with TNF-α stimulation [35]. Accordingly, merging these two lines of evidence, namely that TNF-α is increased in laser choroidal neovascularization and TNF-α enhances FcRn expression, may explain why the laser choroidal neovascularization model has increased FcRn function (Figure 3A). This would translate into more rapid elimination of intravitreally administered IgG into the blood system, which is exactly what we observed in laser CNV model rats (Figure 3B). The inhibition of FcRn upregulation or the FcRn elimination pathway itself may mitigate this increased elimination and thereby enhance the intravitreal half-life of IgG. A method to decrease elimination of IgG from the eye provides a future direction for extending the results of this current study and potentially decreasing the frequency of intraocular IgG injection.

In conclusion, the human blood-ocular barrier expresses the FcRn receptor. In addition, FcRn plays an important role in eliminating intravitreally administered full-length IgGs across the blood-retina barrier into the systemic blood system.
